# Multi-Omics and Machine Learning Identify Immune-Linked Gene Signatures for LUAD Stratification

**DOI:** 10.3390/genes17091096

**Published:** 2026-09-11

**Authors:** Rakesh Arya, Viplov Kumar Biswas, Hemlata Shakya, Moumita Majumdar, Jong-Joo Kim

**Affiliations:** 1 Department of Biotechnology, Yeungnam University, Gyeongsan 38541, Gyeongbuk, Republic of Korea; 2 Department of Veterinary & Biomedical Sciences, University of Minnesota at Twin Cities, 1971 Commonwealth Avenue, Saint Paul, MN 55108, USA; vbiswas@umn.edu; 3 Department of Biomedical Engineering, Shri G. S. Institute of Technology and Science, Indore 452003, Madhya Pradesh, India; hemlata.shakya19@gmail.com; 4 Centre for Scientific Research and Development, People’s University, Bhanpur, Bhopal 462037, Madhya Pradesh, India; moumita.majumdar@peoplesuniversity.edu.in

**Keywords:** lung adenocarcinoma, biomarkers, *CPED1*, single-cell RNA sequencing, prognostic model

## Abstract

**Background**: Lung adenocarcinoma (LUAD) is the most common subtype of non-small-cell lung cancer and is one of the leading causes of cancer-related deaths globally. Despite current developments, reliable biomarkers for effective diagnosis, prognosis, and patient stratification are still lacking. **Methods**: We analyzed publicly available TCGA-LUAD and GEO datasets using integrative bioinformatics approaches, including differential gene expression, weighted gene co-expression network analysis (WGCNA), survival modeling, mutation profiling, immune cell infiltration scores, machine learning, and bulk-RNA and single-cell RNA sequencing. **Results**: A total of 5581 deregulated genes were identified, with the turquoise module (298 genes) showing strong correlation with LUAD (Corr = −0.79, *p* < 2.2 × 10^−308^). The integration of two analyses yielded 281 overlapping genes, out of which nine candidates (*ANO2*, *CHIAP2*, *CPED1*, *DNASE1L3*, *GSTM5*, *HTR3C*, *PRKCE*, *SLC14A1*, and *WNT3A*) were selected via LASSO Cox regression to build a prognostic risk model. High-risk patients have significantly worse survival (log-rank *p* = 0.0027). *CPED1* exhibited the highest mutation frequency, with 41% of TCGA-LUAD samples harboring mutations. Among all *CPED1* mutation events, missense mutations were the most common (47%). GSEA and KEGG analysis revealed significant enrichment of pathways such as nucleocytoplasmic transport, oxidative phosphorylation, protein processing in the endoplasmic reticulum, ribosome, and ribosome biogenesis in high-risk patients. Immune infiltration analysis indicated differences in immune cell infiltration scores between high- and low-immune-score groups, with M1 macrophages showing strong statistical correlation with aDC, monocytes, and CD4^+^ naïve T cells. Machine learning confirmed that the combined Enet+PLS model predicted *CPED1* as a core predictor, and *CPED1* was successfully validated in independent GEO datasets (GSE43458 and GSE31210), showing strong diagnostic accuracy (AUCs up to 0.98). Finally, single-cell RNA sequencing revealed that *CPED1* was mostly expressed in fibroblasts and myeloid cells, with *CPED1* significantly downregulated in LUAD compared with normal samples. **Conclusions**: This study integrates multi-omics and machine learning to highlight *CPED1* as a promising candidate biomarker, with potential diagnostic and prognostic relevance in LUAD. The nine-gene risk signature stratified patients by survival outcomes in the TCGA cohort. Genomic and immune analyses revealed features associated with the high-immune-score group. As the study is entirely computational and the prognostic model lacks external survival validation, these findings should be regarded as preliminary and hypothesis-generating, requiring future independent validation and functional studies to confirm the biological significance and clinical utility of *CPED1* and related genes.

## 1. Introduction

Lung adenocarcinoma (LUAD) poses a serious health concern and is a major cause of death worldwide [1,2]. Non-small-cell lung cancer (NSCLC) accounts for approximately 85% of all lung cancer cases, with LUAD characterized as the most predominant subtype [3,4]. Regardless of recent developments in diagnostic and therapeutic approaches, long-term survival among LUAD patients remains poor, with 5-year survival rates ranging from 4% to 17%, mostly due to late detection, high tumor heterogeneity, and treatment resistance [5]. In 2020, 2.2 million cancer-related cases were documented worldwide, with LUAD comprising a significant proportion, particularly in women, where incidence was reported at 57% [6]. Although targeted therapies and immunotherapies have enhanced treatment options, resistance and relapse remain frequent, highlighting the urgent need for the identification of reliable biomarkers capable of classifying patients by prognosis and therapeutic response to enhance diagnosis, prognosis, and personalized treatment [7].

Because symptoms of lung cancer are not visible in early stages, many patients are diagnosed only at advanced stages, thus complicating treatment response and decreasing survival rate [8]. Current therapeutic strategies including chemotherapy, targeted therapy, and surgery are less effective in advanced disease stages due to the biological heterogeneity of LUAD [9,10]. Despite advances in genomics, immunotherapy, and targeted therapy, the mechanism of LUAD pathogenesis and drug resistance remain incompletely understood. Multi-omics approaches integrating genomics, transcriptomics, and epigenomics have discovered dynamic changes in tumorigenesis and progression, highlighting candidate genes with clinical potential [11]. With the development of precision medicine, statistical and computational machine learning algorithms have been gradually employed to identify new diagnostic and therapeutic targets [12,13].

In this study, we applied integrative bioinformatics approaches to publicly available datasets to identify diagnostic biomarkers. Differential expression analysis and WGCNA identified deregulated genes and modules, with the turquoise module showing the strongest correlation with disease progression. Integration of these results yielded overlapping genes, from which nine candidates were selected via Least Absolute Shrinkage and Selection Operator (LASSO) Cox regression to build a prognostic risk model. Survival analysis showed poor prognosis in high-risk patients, while mutation profiling identified *CPED1* and *ANO2* as the most frequently mutated genes. Pathway enrichment analysis using FDR-adjusted values revealed significant enrichment of nucleocytoplasmic transport, oxidative phosphorylation, protein processing in the endoplasmic reticulum, ribosome, and ribosome biogenesis in high-risk patients. Immune infiltration analysis showed statistical correlations between M1 macrophages and activated dendritic cells, monocytes, and CD4^+^ naïve T cells. Machine learning confirmed *CPED1* as a key predictor, independent GEO validation demonstrated strong diagnostic accuracy, and single-cell RNA showed *CPED1* downregulation in LUAD compared with normal samples. Collectively, these findings highlight *CPED1* as a promising candidate biomarker with potential diagnostic and prognostic relevance in LUAD, while emphasizing the need for future functional and clinical validation to confirm its biological role and clinical utility.

## 2. Materials and Methods

### 2.1. Acquiring Data

**Figure 1:**
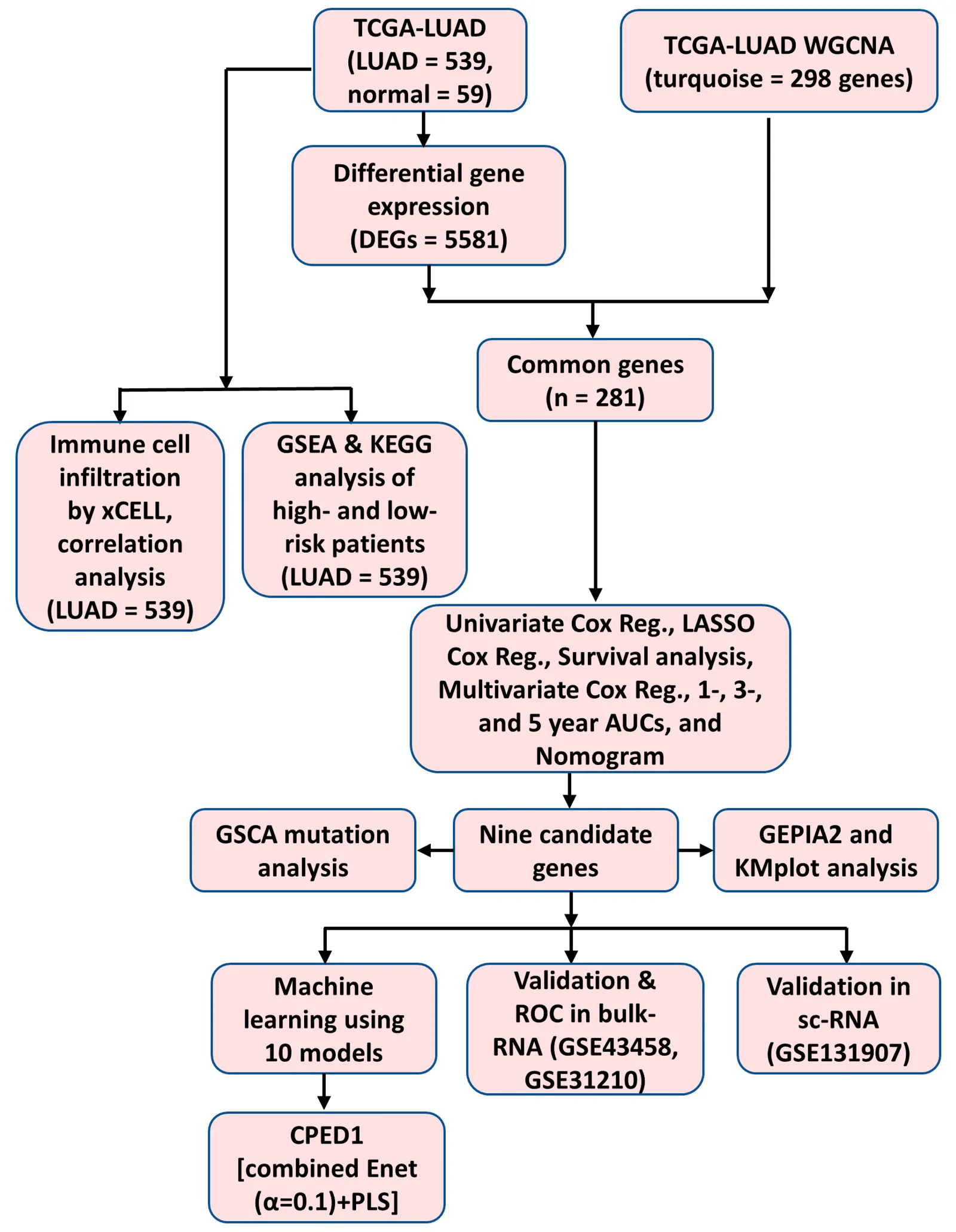
Schematic flow diagram of the integrated bioinformatics pipeline to identify diagnostic and prognostic biomarkers in LUAD.

We downloaded RNA-Seq (STAR-Counts) data comprising 598 samples, including 539 LUAD (primary tumor) and 59 normal (solid tissue normal), using the ‘TCGAbiolinks’ (v2.38.0) package in R language (version 4.5.3) from the Genomic Data Commons (GDC) data portal (https://portal.gdc.cancer.gov/projects/tcga-luad, accessed on 30 June 2025). For validation, we obtained two bulk Gene Expression Omnibus (GEO) datasets: GSE43458 (80 cancer samples and 30 normal samples) [14] and GSE31210 (226 cancer samples and 20 normal samples) [15]. In addition, we downloaded the GSE131907 single-cell RNA-seq dataset (11 cancer samples and 11 normal samples) [16] from the Gene Expression Omnibus (GEO) database (https://www.ncbi.nlm.nih.gov/geo, accessed on 18 August 2025). A schematic flowchart of the methods and results used in this study is shown in Figure 1.

### 2.2. Differential Gene Expression Analysis

Differential gene expression (DEG) analysis between cancer and normal samples in TCGA-LUAD was performed using the ‘DESeq2’ (v1.50.2) package in R. A cutoff of log_2_FC > ±1 and FDR < 0.05 (Benjamini–Hochberg adjustment) was applied to identify significantly differentially expressed genes (DEGs).

### 2.3. Weighted Gene Co-Expression Network Analysis (WGCNA) Crossover Analysis

The ‘WGCNA’ (v1.74) package in R was used to construct and visualize the co-expression network. The top 5000 most variable genes were selected based on variance. A soft threshold of β = 5 (scale-free R^2^ = 0.8) was chosen to construct the network. A cluster dendrogram was generated to visualize modules represented in different colors, and dynamic modules with high resemblances were merged at a cut height of 0.25. Pearson correlation was used to compute the relationships between module eigengenes and clinical traits (normal vs. LUAD). Key LUAD-related genes were selected based on the cutoff for gene significance (GS) > 0.5 and module membership (MM) > 0.7 [17]. Common genes between DEGs and WGCNA were identified using a Venn diagram.

### 2.4. Survival Analysis and Prognostic Model Construction Using TCGA-LUAD Data

This analysis was performed to construct a prognostic risk model to stratify LUAD patients based on gene expression profiles and evaluate survival outcomes. Gene expression and clinical metadata for 539 LUAD patients were retrieved from TCGA using R (v4.5.3). After barcode matching, samples with replicate runs or trimming errors were excluded. Cases with missing or zero survival times were also removed, resulting in a final cohort of 443 LUAD patients for survival analysis. Missing gene values were imputed with median substitution and normalized with z-score scaling. Univariate Cox regression analysis of 281 common genes was performed using the ‘survival’ (v3.8.9) package, and genes with *p* < 0.05 were retained. Significant genes were visualized in forest plots using ‘forestplot’ (v3.2.0), with the top 10 genes highlighted. Feature selection was performed using LASSO Cox regression with Elastic Net regularization (α = 0.2), corresponding to an L1 ratio of 0.2 (20% lasso and 80% ridge penalty), and 10-fold cross-validation via ‘glmnet’ (v5.0), with stratified folds generated by ‘caret’ (v7.0.1). This identified nine prognostic genes, which were used to construct the risk score formula:

$$Risk\;score=\sum_{i=1}^{n}\beta_{i}\times Expression({Gene}_{i}),$$

where *β*_*i*_ represents the regression coefficient of each gene. Patients (*n* = 443) were divided into high- and low-risk groups according to the nine-gene median prognostic risk score as the cutoff, with individuals above the median classified as high-risk and those below classified as low-risk. Kaplan–Meier curves and log-rank tests were created with ‘survminer’ (v0.5.2) and ‘ggpubr’ (v1.0.0). Predictive accuracy was assessed by time-dependent ROC curves at 1, 3, and 5 years using ‘timeROC’ (v0.4.1), based on all 443 patients. Multivariate Cox regression integrating gender, AJCC pathological stage, and risk score was performed on 379 patients with complete stage information, and results were visualized in forest plots. Finally, a nomogram assessing survival probabilities at 1, 3, and 5 years was constructed using ‘rms’ (v8.1.1) based on the same 379 patients with complete clinical data.

### 2.5. GEPIA2 and KM-Plot Analysis

Gene expression profiling interactive analysis (GEPIA2, http://gepia2.cancer-pku.cn, accessed on 15 June 2026) was used to compare expression levels of candidate genes between LUAD (*n* = 483) and normal samples (*n* = 347) using default parameters (*p* = 0.05, log_2_ (TPM+1)) [18]. For survival analysis, the KM-Plot tool (https://kmplot.com/analysis/index.php?p=service&cancer=lung, accessed on 15 June 2026) was employed to generate Kaplan–Meier curves and log-rank tests, stratifying patients into high- and low-expression groups.

### 2.6. Mutation Analysis

Mutation frequency, copy number variations (CNVs), and mutation type of candidate genes in LUAD were analyzed using the Gene Set Cancer Analysis (GSCA) database https://guolab.wchscu.cn/GSCA/#/, accessed on 15 June 2026). Mutation frequencies were calculated using the TCGA-LUAD cohort (*n* = 567 tumor samples) as the denominator. CNVs were analyzed separately to distinguish amplification and deletion events. Mutation type distributions were reported as proportions of mutation events within each gene.

### 2.7. Gene Set Enrichment Analysis (GSEA) and KEGG Pathways of DEGs Between High- and Low-Risk Patients

Transcriptomic data from 539 TCGA-LUAD samples were stratified into high- and low-risk groups using the median risk score as the cutoff. Differentially expressed genes between the two groups were identified using the’ limma’ package (v3.60.6) in R, applying moderated t-statistics. Gene symbols were converted to Entrez IDs using the ‘org.Hs.eg.db’ package (v3.20.0) and the ‘bitr’ function. GSEA was performed using the “clusterProfiler” package (v4.18.4), using the fgsea algorithm with increased permutations (nPermSimple = 100,000) to stabilize *p*-value estimation. Importantly, enrichment was evaluated using false discovery rate (FDR)-adjusted *p*-values (p.adjust), rather than nominal *p*-values, to control for multiple testing across pathways. Visualization included enrichment plots for the top five KEGG pathways ranked by FDR values, and bubble plots summarizing the top twenty pathways. In both plots, statistical significance was represented by −log10 (FDR) values, ensuring that pathway prioritization reflected multiple-testing correction.

### 2.8. Immune Cell Infiltration and Correlation Analysis

This analysis was performed to investigate differences in immune cell infiltration between high- and low-immune-score LUAD groups and to explore associations among immune populations within the tumor microenvironment. Transcriptomic data from 539 LUAD samples were analyzed using the ‘xCell’ (v1.1.0) algorithm to estimate immune cell infiltration. A panel of 20 immune cell types was profiled, and patients were stratified into high- and low-immune-score groups according to the summed immune-cell scores derived from xCell, using the median score as the cutoff. This stratification was used to explore differences in the tumor microenvironment. Visualization included stacked bar plots to show relative immune composition and boxplots to compare distributions. Statistical testing was performed using the Wilcoxon rank-sum test to identify significantly different cell types between high- and low-immune-score groups. Statistical correlations were performed between M1 macrophages and significantly associated immune populations.

### 2.9. Machine Learning Model Construction and Gene Selection

Gene selection and classification performance were evaluated using multiple machine learning algorithms implemented in R (v4.5.3). Expression data from independent GEO cohorts (GSE43458 as the training set and GSE31210 as the test set) were standardized by z-score scaling, using training set statistics only. Eight common candidate genes, excluding *WNT3A* (which was missing from the GSE31210 dataset), were used as input features. Models included Random Forest (RF; v4.7.1.2), eXtreme Gradient Boosting (XGB; v3.2.1.1), Elastic Net (Enet; α = 0.1–0.9, glmnet v5.0), Support Vector Machine-Recursive Feature Elimination (SVM-RFE; e1071 v1.7.17), Partial Least Squares (PLS; plsRglm v1.7.1), Linear Discriminant Analysis (LDA; caret v7.0.1), Naïve Bayes (NB; e1071 v1.7.17), glmBoost (mboost v2.9.11), Stepwise GLM (StepGLM; caret v7.0.1), and K-Nearest Neighbors (KNN; caret v7.0.1). For KNN, dimensionality reduction was performed using principal component analysis (PCA) restricted to the common genes, with six principal components retained. Models were trained by repeated 10-fold cross-validation using the ‘caret’ framework, optimizing for ROC performance. The KNN classifier was tuned over k = 4–9 neighbors. Weighted ensemble models were constructed by combining predictions from top-performing algorithms. External validation was performed on the independent test set, with predictive accuracy assessed by ROC curves and AUC values. Gene importance was extracted from the trained models, and results were visualized using bar plots for interpretability.

### 2.10. Validation of Candidate Genes in Independent Bulk RNA-Seq Datasets

Expression levels of the candidate genes were validated using two independent GEO bulk RNA-seq datasets: GSE43458 and GSE31210. Boxplots were generated to visualize the log_2_-transformed expression differences between normal and LUAD samples. Statistical significance between groups was assessed using the Wilcoxon rank-sum test (nominal *p*-values). Receiver operating characteristic (ROC) curve analysis was performed to evaluate the diagnostic performance, with area under the curve (AUC) values calculated. The ROC plots were generated using SRplot (https://bioinformatics.com.cn/, accessed on 15 June 2026).

### 2.11. Single-Cell RNA Sequencing Analysis

This analysis introduced an independent single-cell RNA-seq dataset to validate candidate gene expression at the cellular level, providing finer resolution of cell-type-specific patterns beyond bulk RNA-seq. Single-cell RNA sequencing data from 11 LUAD and 11 normal lung tissues from the GSE131907 scRNA dataset were processed in R (v4.5.3) using Seurat (v5.5.1). Raw UMI matrices were loaded and a Seurat object was created with thresholds of min.cells = 10 and min.features = 300. Quality control metrics included mitochondrial gene percentage (percent.mt) and hemoglobin gene percentage (percent.hb). Cells were filtered using the following criteria: nFeature_RNA 200–5000, nCount_RNA 500–30,000, percent.mt < 10%, and percent.hb < 5%. Normalization was performed using LogNormalize (scale factor = 10,000), and highly variable genes (HVGs) were identified via the VST method (top 2000 features). Data were scaled on HVGs, followed by principal component analysis (PCA, 50 PCs). Dimensionality reduction was refined with Harmony batch correction. The number of significant PCs was determined using JackStraw (50 replicates, 20 PCs), ElbowPlot, and variance explained plots.

Clustering was performed with FindNeighbors (dims = 1–40, k.param = 20) and FindClusters (resolution = 0.2, Louvain algorithm), followed by UMAP embedding (dims = 1–40, min.dist = 0.5, spread = 1.5, n.neighbors = 30). Marker genes for each cluster were identified using FindAllMarkers (test.use = “wilcox”, min.pct = 0.25, logfc.threshold = 0.25, max.cells.per.ident = 400), with significant markers defined at adjusted *p* < 0.05. Heatmaps were generated for the top 20 marker genes per cluster.

Cell type annotation was performed based on canonical marker genes [16,19]. Major identified cell types included T cells (CD3D, CD3E, CD3G, TRAC), B cells (CD79A, IGHM, IGHG3, IGHA2), NK cells (NKG7, GNLY, KLRD1), mast cells (KIT, MS4A2, GATA2), myeloid cells (CD68, MARCO, LYZ), fibroblasts (DCN, COL1A1, COL1A2), endothelial cells (PECAM1, CLDN5, RAMP2), and epithelial cells (EPCAM, KRT19, CDH1, KRT18). These annotations were combined into the Seurat metadata to enable downstream comparisons between LUAD and normal samples.

To ensure statistical robustness, we applied pseudobulk analysis, aggregating cell-level counts into sample-level profiles (11 LUAD vs. 11 normal biological replicates). This approach avoids inflated significance that can arise when treating thousands of cells as independent replicates. Finally, the expression of eight candidate genes (*ANO2*, *CPED1*, *DNASE1L3*, *GSTM5*, *HTR3C*, *PRKCE*, *SLC14A1*, and *WNT3A*) was systematically validated across annotated cell types and between normal and LUAD groups. Statistical testing of candidate gene expression between normal and LUAD samples was performed using the Wilcoxon rank-sum test on pseudobulk (sample-level) averages. *p*-values were adjusted for multiple testing using the Benjamini–Hochberg false discovery rate (FDR) method; genes with an FDR < 0.05 were considered significant. Visualization was performed using dot plots, feature plots, violin plots, and box plots to highlight distribution patterns across cell types.

## 3. Results

### 3.1. Differential Expression and WGCNA of LUAD Genes

**Figure 2:**
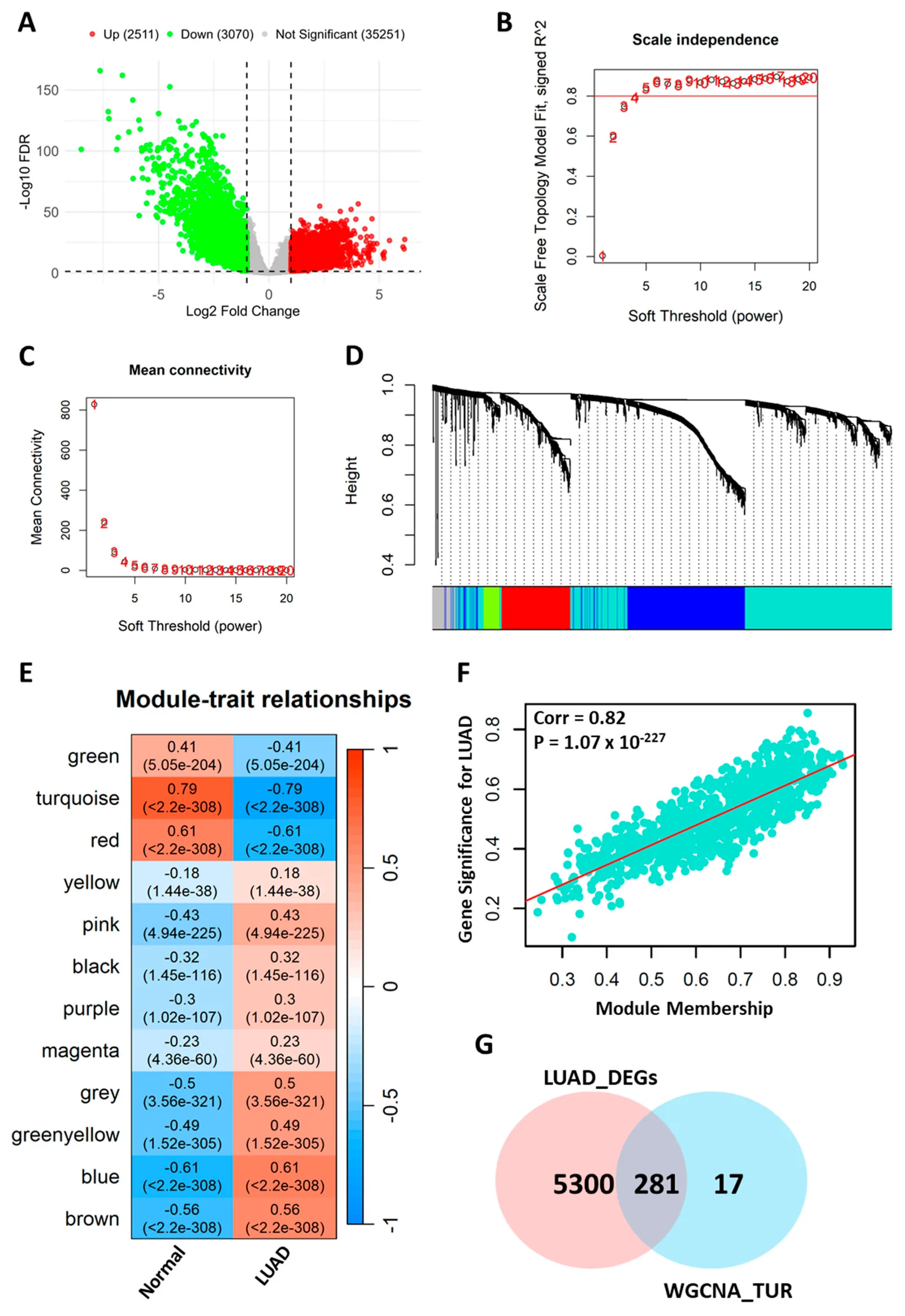
Differential expression and weighted gene co-expression network analysis (WGCNA) for LUAD. (**A**) Volcano plot showing 2511 upregulated genes (red), 3070 downregulated genes (green), and 35,251 non-significant genes (gray) identified using log2FC > ±1 and FDR < 0.05. (**B**) Scale-free topology model fit (signed R^2^) plotted against soft-thresholding power, with stabilization at β = 5. (**C**) Mean connectivity values plotted against soft-thresholding power. (**D**) Hierarchical clustering dendrogram with modules represented in distinct colors. (**E**) Heatmap showing correlations between gene modules and clinical traits (Normal vs. LUAD), with correlation coefficients and *p*-values. (**F**) Scatter plot of turquoise module membership (MM) versus gene significance (GS). (**G**) Venn diagram showing overlap of 281 genes between LUAD_DEGs (total differentially expressed genes in TCGA-LUAD dataset) and WGCNA_TUR (genes identified in the turquoise module using WGCNA).

Differential expression analysis identified a total of 5581 genes (Table S1), of which 2511 were upregulated and 3070 were downregulated in LUAD (*n* = 539) compared with normal tissues (*n* = 59), with 35,251 genes showing no significant change (Figure 2A). WGCNA was performed to construct modules of co-expressed genes. The scale-free topology fit index increased with higher soft-thresholding power, stabilizing at an optimal threshold value of β = 5 (Figure 2B), while mean connectivity decreased accordingly (Figure 2C). Hierarchical clustering identified multiple gene modules, with major clusters represented by colors such as turquoise, blue, and red, each corresponding to sets of co-expressed genes used for downstream trait correlation analysis (Figure 2D), which were subsequently correlated with clinical traits.

Module–trait relationship analysis revealed that the turquoise module (*n* = 298; Table S2) was strongly correlated with the LUAD group (Corr = −0.79, *p* < 2.2 × 10^−308^), suggesting its potential relevance to disease progression (Figure 2E). Therefore, we selected the turquoise module as the target module for analysis. By introducing module membership (MM) and gene significance (GS), we examined the correlation between genes and phenotypes within the turquoise module and generated an MM–GS correlation scatter plot (Figure 2F). The results showed a significant positive correlation between GS and MM (Corr = 0.82, *p* = 1.07 × 10^−227^), meaning that genes most strongly associated with LUAD status are also the most central to the turquoise module. Finally, integration of differentially expressed LUAD genes with the turquoise module identified 281 overlapping genes (Figure 2G), suggesting that these genes may represent biologically meaningful drivers of LUAD.

### 3.2. Prognostic Gene Signature and Survival Modeling

**Figure 3:**
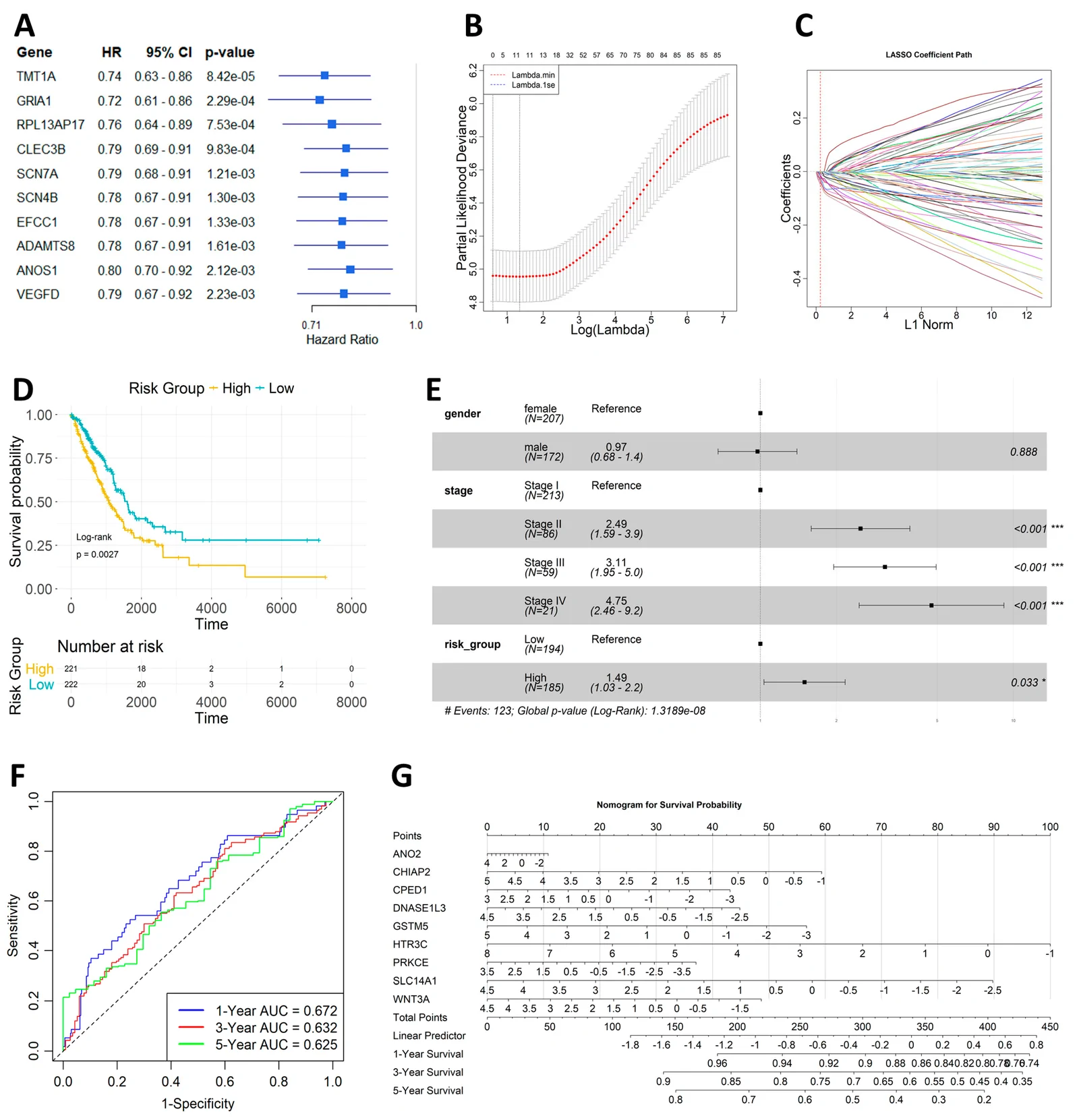
Construction and validation of the LUAD prognostic gene signature. (**A**) Forest plot of univariate Cox regression results for 281 genes, with hazard ratios and confidence intervals shown for the top 10 genes. (**B**) Partial likelihood deviance curve showing optimal lambda values used for LASSO Cox regression. (**C**) LASSO coefficient paths for genes along the regularization path, with nine genes retained at the optimal lambda. (**D**) Kaplan–Meier survival curves comparing high- and low-risk groups defined by the median risk score cutoff. (**E**) Multivariate Cox regression forest plot showing hazard ratios and 95% confidence intervals for gender, AJCC pathological stage, and the nine-gene risk group. The vertical dashed line indicates HR = 1. Significance levels: * *p* < 0.05, *** *p* < 0.001. (**F**) Time-dependent ROC curves at 1, 3, and 5 years. (**G**) Nomogram integrating the nine-gene risk score with clinical variables to predict survival probabilities at 1, 3, and 5 years.

Out of 281 common genes, univariate Cox regression identified 86 genes significantly linked with LUAD prognosis (*p* < 0.05). The top 10 significantly associated genes, with hazard ratios and confidence intervals, are presented in Figure 3A. These 86 genes were then subjected to LASSO Cox regression with 10-fold cross-validation to perform feature selection. Figure 3B shows the partial likelihood deviance curve, from which the optimal lambda value was determined, and Figure 3C illustrates how gene coefficients changed along the regularization path. At the optimal lambda, nine genes (*ANO2*, *CHIAP2*, *CPED1*, *DNASE1L3*, *GSTM5*, *HTR3C*, *PRKCE*, *SLC14A1*, and *WNT3A*) were retained to construct the prognostic risk score model. The expression levels of these nine genes were weighted by their regression coefficients to generate the risk score formula, as follows: Risk score = (−0.158020245 × Expression (*ANO2*)) + (−0.254479447 × Expression (*CHIAP2*)) + (−0.169708368 × Expression (*CPED1*)) + (−0.215797563 × Expression (*DNASE1L3*)) + (−0.213056858 × Expression (*GSTM5*)) + (−0.250341356 × Expression (*HTR3C*)) + (−0.178339343 × Expression (*PRKCE*)) + (−0.226503916 × Expression (*SLC14A1*)) + (−0.192968544 × Expression (*WNT3A*)). Patients (*n* = 443) were stratified into high-risk and low-risk groups according to the median risk score. The regression coefficients, hazard ratios, confidence intervals, and *p*-values for all nine genes are provided in Table S3. Kaplan–Meier analysis demonstrated that patients stratified by the median risk score cutoff had significantly different survival outcomes, with the high-risk group showing worse survival compared with the low-risk group (log-rank *p* = 0.0027; Figure 3D).

Multivariate Cox regression incorporating gender, AJCC pathological stage, and risk score was performed on 379 patients with complete stage information. This analysis confirmed that the nine-gene risk score was an independent prognostic factor after adjusting for clinical variables (HR = 1.43, 95% CI = 1.03–2.20, *p* = 0.033; Figure 3E, Table S4). Time-dependent ROC curves showed predictive accuracy, with AUCs of 0.672, 0.632, and 0.625 at 1, 3, and 5 years, respectively, based on all 443 patients (Figure 3F). Among the nine genes, *CPED1*, *PRKCE*, and *ANO2* showed stronger associations with survival risk, while *DNASE1L3* and *GSTM5* exhibited weaker prognostic influence. Finally, a nomogram was constructed using 379 patients with complete clinical data, integrating the nine-gene risk score with clinical variables to predict 1-year, 3-year, and 5-year survival probabilities. Higher total points corresponded to reduced survival likelihood (Figure 3G).

### 3.3. Gene Expression and Prognostic Value in LUAD

**Figure 4:**
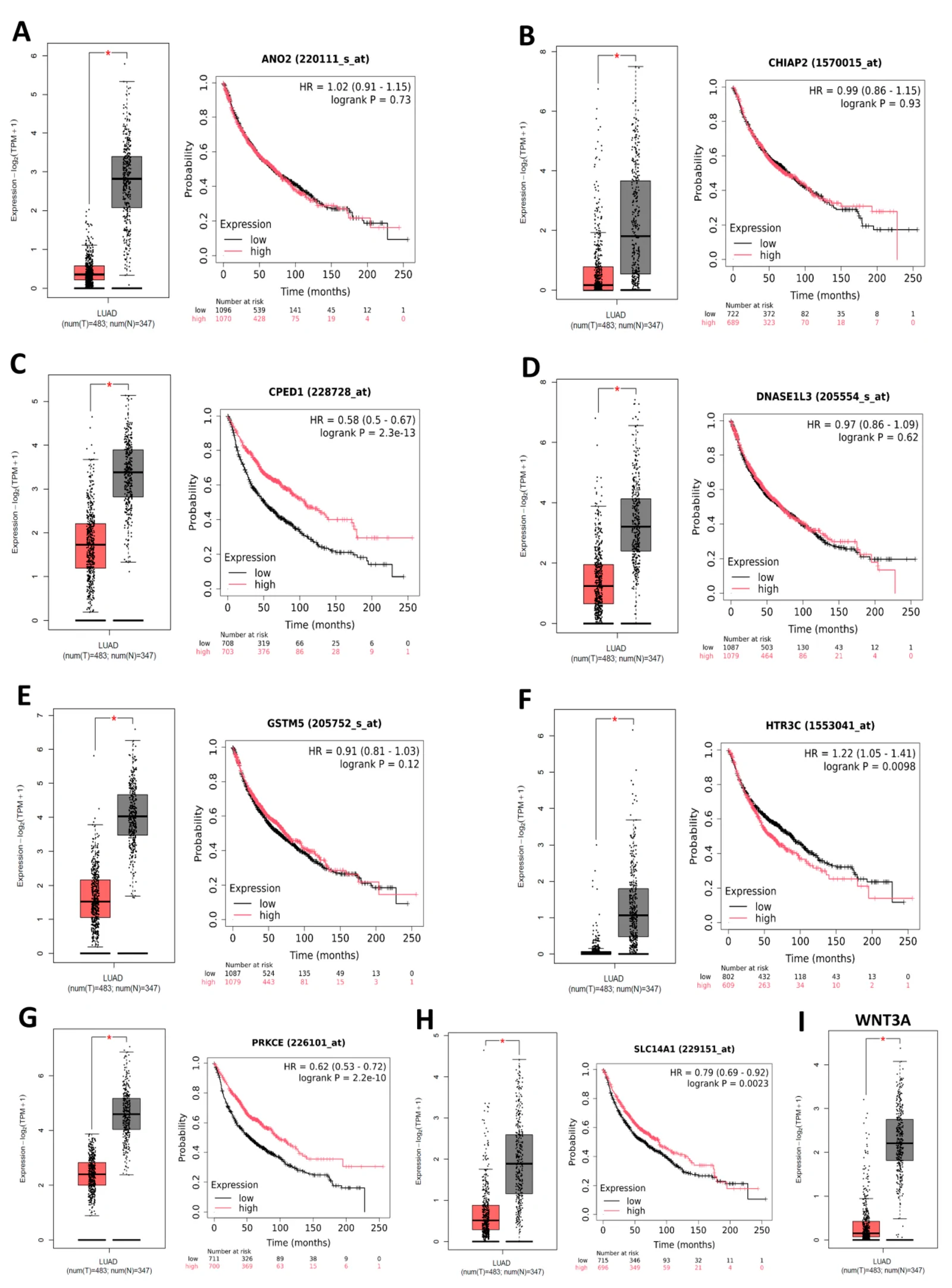
Gene expression and survival analysis of LUAD candidate genes. (**A**–**I**) Boxplots (**left**) show gene expression differences between LUAD (*n* = 483) and normal samples (*n* = 347), while Kaplan–Meier curves (**right**) display survival probabilities for patients stratified by high versus low expression of each gene. Red asterisks in the boxplots indicate statistically significant differences between LUAD and normal groups (*p* < 0.05).

Expression analysis from the GEPIA2 database revealed significant differences between LUAD and normal samples for the nine candidate genes (Figure 4A–I). Survival associations were evaluated for eight genes using KM-Plot: *ANO2* (HR = 1.02, *p* = 0.73), *CHIAP2* (HR = 0.99, *p* = 0.93), *CPED1* (HR = 0.58, *p* = 2.3 × 10^−13^), *DNASE1L3* (HR = 0.97, *p* = 0.62), *GSTM5* (HR = 0.91, *p* = 0.12), *HTR3C* (HR = 1.22, *p* = 0.0098), *PRKCE* (HR = 0.62, *p* = 2.2 × 10^−10^), and *SLC14A1* (HR = 0.79, *p* = 0.0023) were assessed for associations with overall survival. *CPED1*, *PRKCE*, and *SLC14A1* emerged as protective factors, while *HTR3C* was linked to poorer prognosis. The remaining genes (*ANO2*, *CHIAP2*, *DNASE1L3*, *GSTM5*) did not show significant survival associations (*p* > 0.05). Data for *WNT3A* were not available in the KM-Plot database.

### 3.4. Mutation Frequencies and Alteration Types in LUAD Candidate Genes

**Figure 5:**
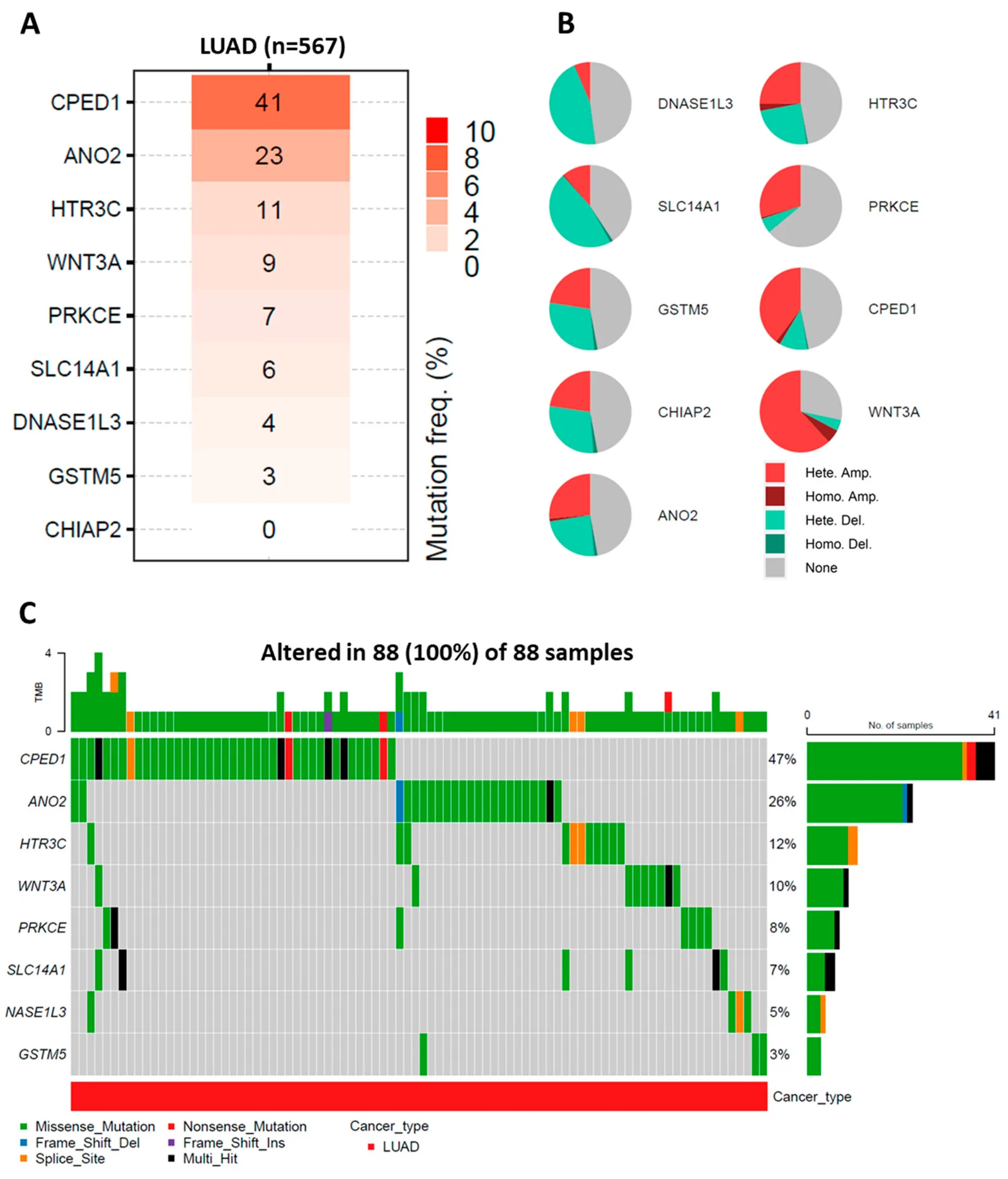
Mutation frequencies and alteration types of LUAD candidate genes. (**A**) Heatmap showing mutation frequencies (%) across nine candidate genes, calculated using TCGA-LUAD tumor samples (*n* = 567) as the denominator, with *CPED1* showing the highest mutation frequency (41% of samples). (**B**) Pie charts illustrating copy number variation (CNV) patterns (heterozygous/homozygous amplification or deletion) for each gene. (**C**) Bar plot summarizing mutation type distribution within mutated samples, showing that missense mutations accounted for 47% of all *CPED1* mutation events.

Mutation analysis of nine candidate genes revealed variable alteration frequencies across LUAD samples. *CPED1* exhibited the highest mutation frequency, with 41% of TCGA-LUAD tumor samples (*n* = 567) harboring at least one mutation event. *ANO2* showed the second highest mutation frequency (23%), followed by *HTR3C* (11%), *WNT3A* (9%), *PRKCE* (7%), *SLC14A1* (6%), *DNASE1L3* (4%), and *GSTM5* (3%), while *CHIAP2* exhibited no detectable mutations (Figure 5A). Copy number variation (CNV) analysis specified heterogeneous patterns of amplification and deletion across these genes. *CPED1* and *WNT3A* were mostly affected by amplifications, whereas *DNASE1L3* and *SLC14A1* showed frequent deletions (Figure 5B). Mutation type distribution analysis confirmed that missense mutations were the most common, accounting for 47% of all *CPED1* mutation events and 26% of *ANO2* mutation events (Figure 5C). Thus, the 41% value reflects the proportion of LUAD samples carrying *CPED1* mutations, while the 47% value reflects the proportion of missense mutations among all *CPED1* mutation events.

### 3.5. GSEA Pathway Enrichment Analysis in High- and Low-Risk LUAD Patients

**Figure 6:**
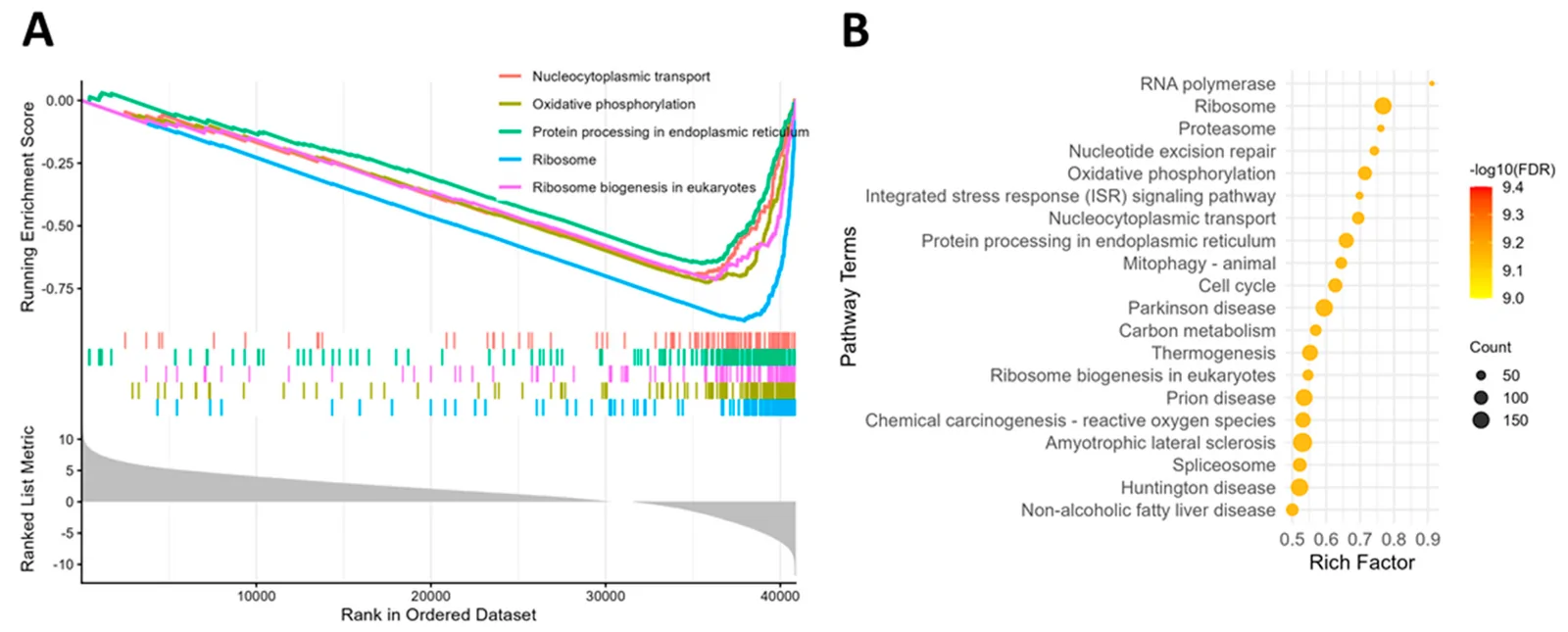
Potential pathway enrichment analysis in high- versus low-risk LUAD patients (*n* = 539). (**A**) GSEA enrichment plots showing the top five KEGG pathways ranked by FDR-adjusted *p*-values enriched in high-risk patients. (**B**) Bubble plot of the top twenty KEGG pathways ordered by FDR values, where circle size indicates the number of core genes, and color represents −log10 (FDR) significance.

GSEA pathway enrichment analysis discovered that several biological processes were significantly enriched in the high-risk group after FDR correction, including nucleocytoplasmic transport, oxidative phosphorylation, protein processing in the endoplasmic reticulum, ribosome, and ribosome biogenesis in eukaryotes (Figure 6A). Statistical analysis of KEGG enrichment confirmed that these pathways remained significant when ranked by FDR-adjusted *p*-values, with bubble plots highlighting additional processes such as RNA polymerase, proteasome, nucleotide excision repair, and neurodegenerative disease pathways (Figure 6B).

### 3.6. Immune Cell Infiltration Between High- and Low-Immune-Score Groups

**Figure 7:**
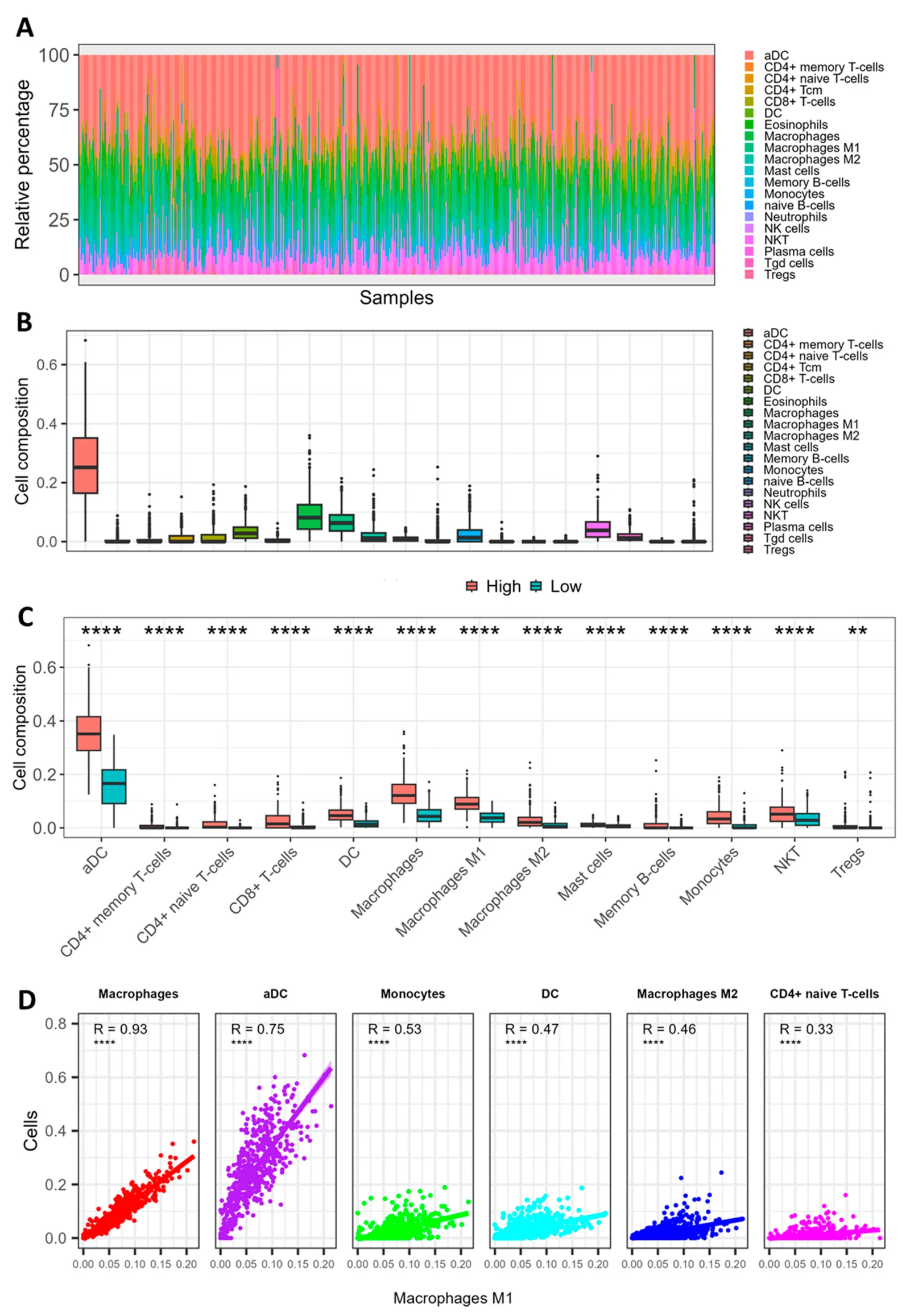
Immune cell composition and correlations in high- and low-immune-score LUAD groups. (**A**) Stacked bar plot showing relative proportions of 20 immune cell types across LUAD samples (*n* = 539). (**B**) Boxplots showing tumor microenvironment (TME) cell composition across samples. (**C**) Boxplots comparing immune cell distributions between high- and low-immune-score groups. Significance was determined using Wilcoxon tests with FDR-adjusted *p*-values; asterisks indicate corrected thresholds (** FDR < 0.01, **** FDR < 0.0001). (**D**) Scatterplots showing correlations between M1 macrophages and other immune populations, with Pearson correlation coefficients (R) and significance levels based on FDR-adjusted *p*-values.

Immune infiltration analysis revealed differences in immune cell composition between high- and low-immune-score groups. The stacked bar plot demonstrates variable proportions of 20 immune cell types across samples (Figure 7A). Boxplot analysis shows that activated dendritic cells were the most abundant population, followed by macrophages, M1 macrophages, and natural killer T cells (NKTs) (Figure 7B). Patients were classified into high- and low-immune-score groups, and several immune cell types were significantly different between groups based on FDR-adjusted *p*-values. Activated dendritic cells, macrophages, M1 macrophages, M2 macrophages, monocytes, and NKTs were significantly higher in the high-immune-score group (FDR < 0.05) (Figure 7C). Correlation analysis identified M1 macrophages as a central hub correlated with multiple immune populations (Figure S1). The strongest correlation was observed with total macrophages (R = 0.93, FDR < 0.0001), followed by aDC (R = 0.75, FDR < 0.0001), monocytes (R = 0.53, FDR < 0.0001), DC (R = 0.47, FDR < 0.0001), macrophages M2 (R = 0.46, FDR < 0.0001), and CD4^+^ naïve T cells (R = 0.33, FDR < 0.0001) (Figure 7D).

### 3.7. Model Performance and Gene Importance in LUAD

**Figure 8:**
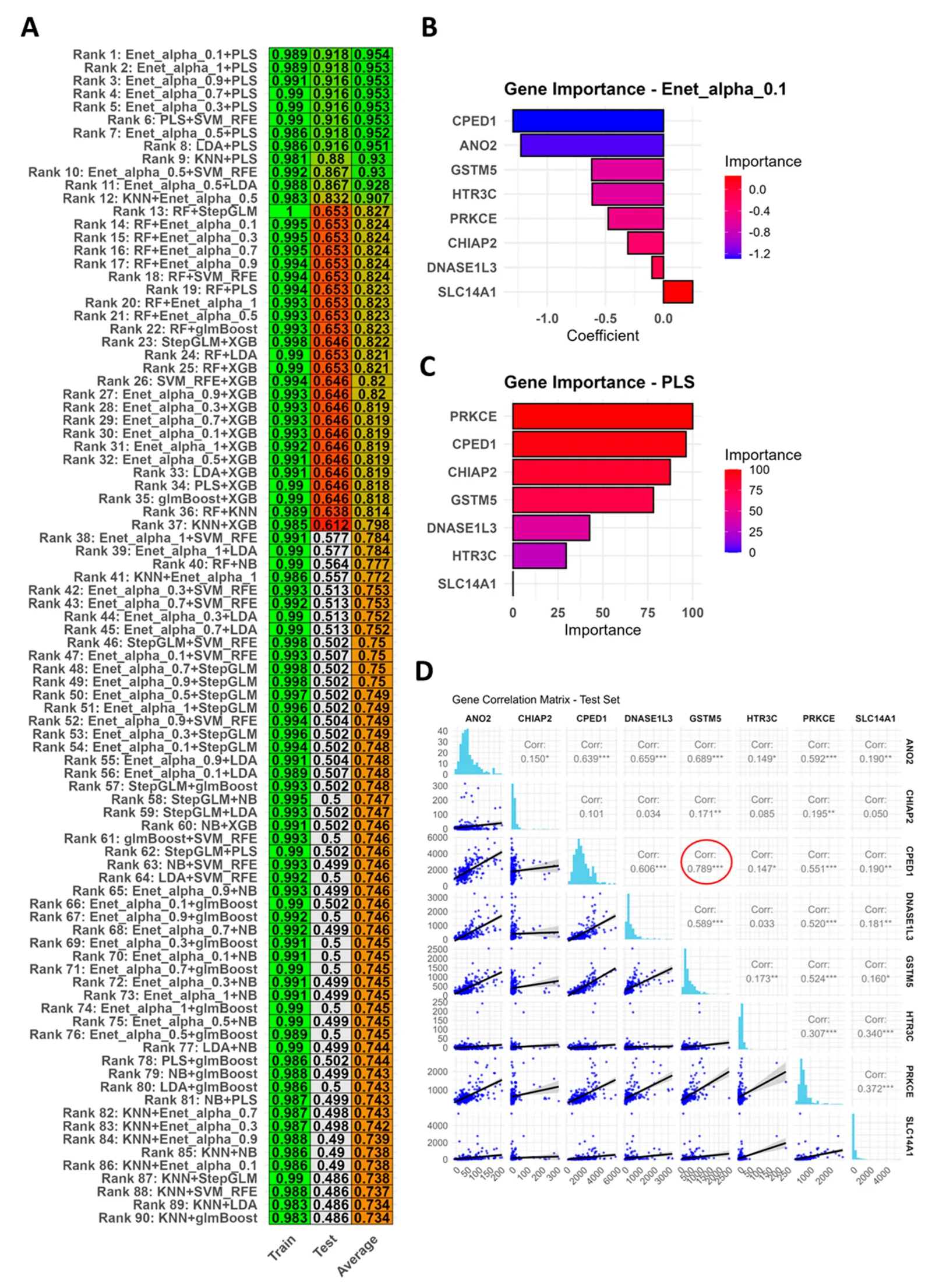
Machine learning model performance and gene importance in LUAD. (**A**) Heatmap showing comparative accuracy of predictive models (Enet, PLS, RF, XGB, SVM-RFE, LDA, NB, glmBoost, stepGLM, and KNN). (**B**) Horizontal bar plots showing gene importance coefficients from Enet (α = 0.1). (**C**) Horizontal bar plot showing gene importance scores from PLS analysis. (**D**) Gene correlation matrix in the test set, showing pairwise associations among candidate genes. The red circle highlights the strongest positive correlation (*CPED1* vs. *GSTM5*, Corr = 0.789). Significance levels are indicated as * *p* < 0.05, ** *p* < 0.01, and *** *p* < 0.001.

To evaluate diagnostic accuracy, several machine learning models were compared across training (GSE43458) and testing (GSE31210) datasets. Eight genes, excluding *WNT3A* (which was missing in the GSE31210 dataset), were used for this analysis. About 90 combined diagnostic models were fitted using machine learning algorithms. Elastic Net (Enet), with α = 0.1, achieved the highest average accuracy in combination with PLS, outperforming RF, XGB, SVM-RFE, LDA, NB, glmBoost, stepGLM, and KNN approaches (Figure 8A). Gene importance analysis revealed that *CPED1*, *ANO2*, *GSTM5*, *HTR3C*, *PRKCE*, *CHIAP2*, *DNASE1L3*, and *SLC14A1* contributed significantly to model performance, with *CPED1* and *ANO2* showing strong coefficients in Enet (α = 0.1), while PLS highlighted *PRKCE* and *CPED1* as key predictors (Figure 8B,C).

Correlation analysis of the testing dataset highlighted associations among eight genes, with the highest correlation of *CPED1* with *GSTM5* (Corr = 0.789, *p* < 0.001), suggesting potential co-regulation patterns within LUAD biology (Figure 8D). These findings underscore the robustness of the combined Enet (α = 0.1) + PLS model and highlight *CPED1* as a potential gene for diagnostic prediction.

### 3.8. Validation and Diagnostic Potential of Candidate Genes in LUAD

**Figure 9:**
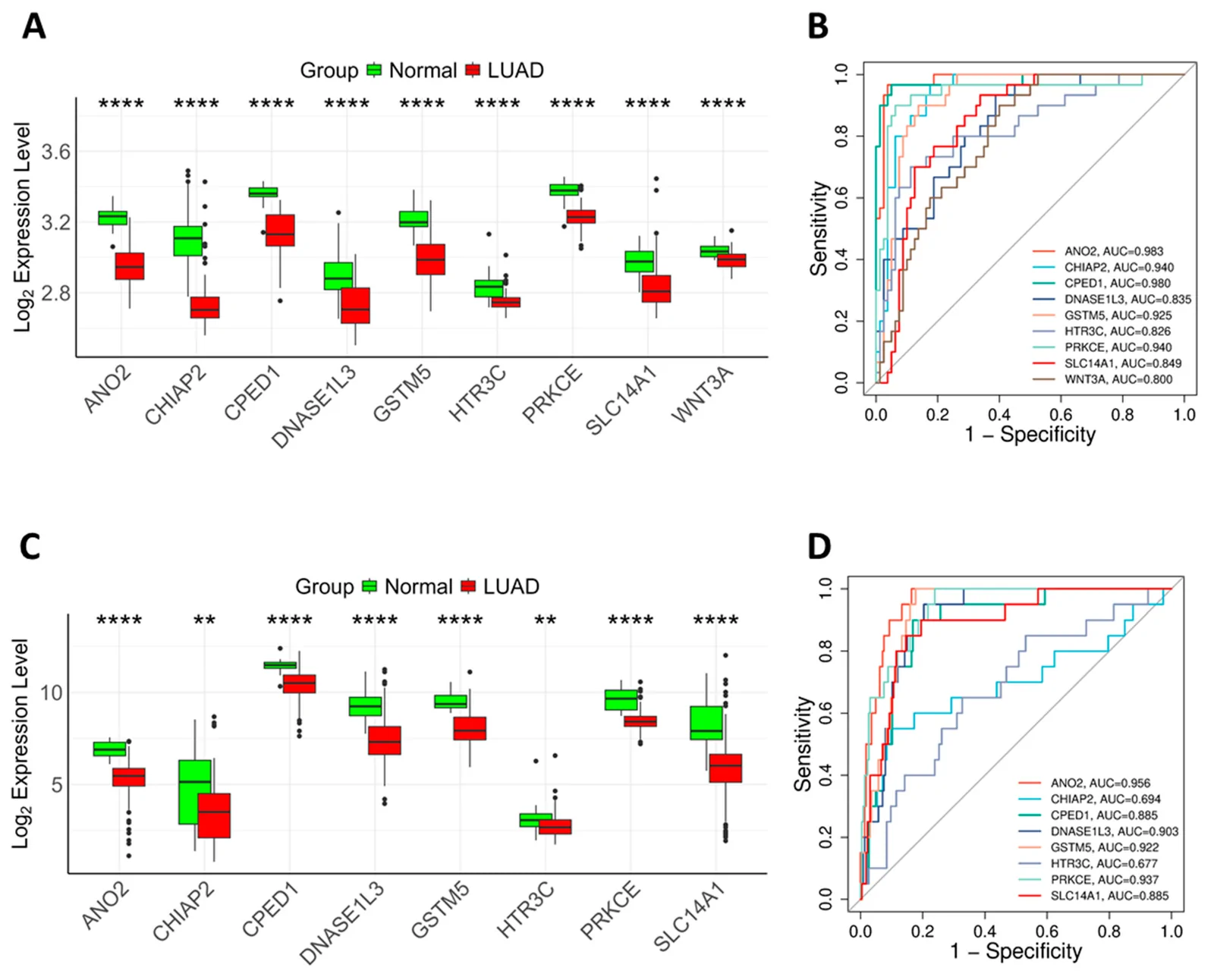
Validation and diagnostic performance of candidate genes in independent bulk RNA-seq datasets. Boxplots show log_2_-transformed expression differences in candidate genes between LUAD and normal samples in (**A**) GSE43458 and (**C**) GSE31210 datasets. Statistical significance was determined by Wilcoxon rank-sum test (nominal *p*-values; ** *p* < 0.01, **** *p* < 0.0001). ROC curves displaying the diagnostic performance of candidate genes in (**B**) GSE43458 and (**D**) GSE31210 datasets.

In the GSE43458 dataset, expression analysis revealed significant differences between normal and LUAD samples for nine candidate genes (nominal *p* < 0.05; Figure 9A). ROC analysis showed that *ANO2* (AUC = 0.983), *CPED1* (AUC = 0.980), *CHIAP2* (AUC = 0.940), *PRKCE* (AUC = 0.940), and *GSTM5* (AUC = 0.925) demonstrated excellent discriminatory ability (Figure 9B). In the GSE31210 dataset, eight genes displayed significant differences between normal and LUAD samples (*WNT3A* was not detected in this cohort) (nominal *p* < 0.05; Figure 9C). ROC analysis indicated that *ANO2* (AUC = 0.956), *PRKCE* (AUC = 0.937), *GSTM5* (AUC = 0.922), *DNASE1L3* (AUC = 0.903), *CPED1* (AUC = 0.885), and *SLC14A1* (AUC = 0.885) maintained strong diagnostic accuracy (Figure 9D).

### 3.9. Single-Cell Landscape and Gene Expression Differences in LUAD

To characterize LUAD cellular heterogeneity, we performed single-cell transcriptomic profiling on 270,901 cells from 11 LUAD and 11 normal samples in the GSE131907 dataset, using eight genes for analysis after excluding *CHIAP2*, which was missing from the dataset. After quality control filtering, 94,791 high-quality cells expressing 25,435 unique genes were retained. Correlation analysis showed a weak association between sequencing depth and mitochondrial gene percentage (r = 0.24) (Figure S2A). In contrast, sequencing depth and number of genes demonstrated a strong positive correlation (r = 0.92) (Figure S2B). The sequencing depth distribution across 22 samples was consistent, with most cells expressing 500–5000 genes (Figure S2C). Gene count distributions confirmed adequate transcript capture across samples (Figure S2D), and direct comparison of sequencing depth and gene counts validated dataset reliability (Figure S2E). Violin plots further illustrated variability across samples: nFeature_RNA ranged from 200 to 5000 per cell, nCount_RNA ranged from 500 to 30,000, and percent.mt remained below 10%, confirming high-quality data suitable for downstream single-cell analysis in LUAD. A total of 2000 highly variable genes (HVGs) across cells, including representative genes such as IGLC2 and IGLC3, were identified to capture biological heterogeneity (Figure S2F). PCA based on HVGs yielded 20 PCs, which captured transcriptomic variation in LUAD and normal cells (Figure S2G).

**Figure 10:**
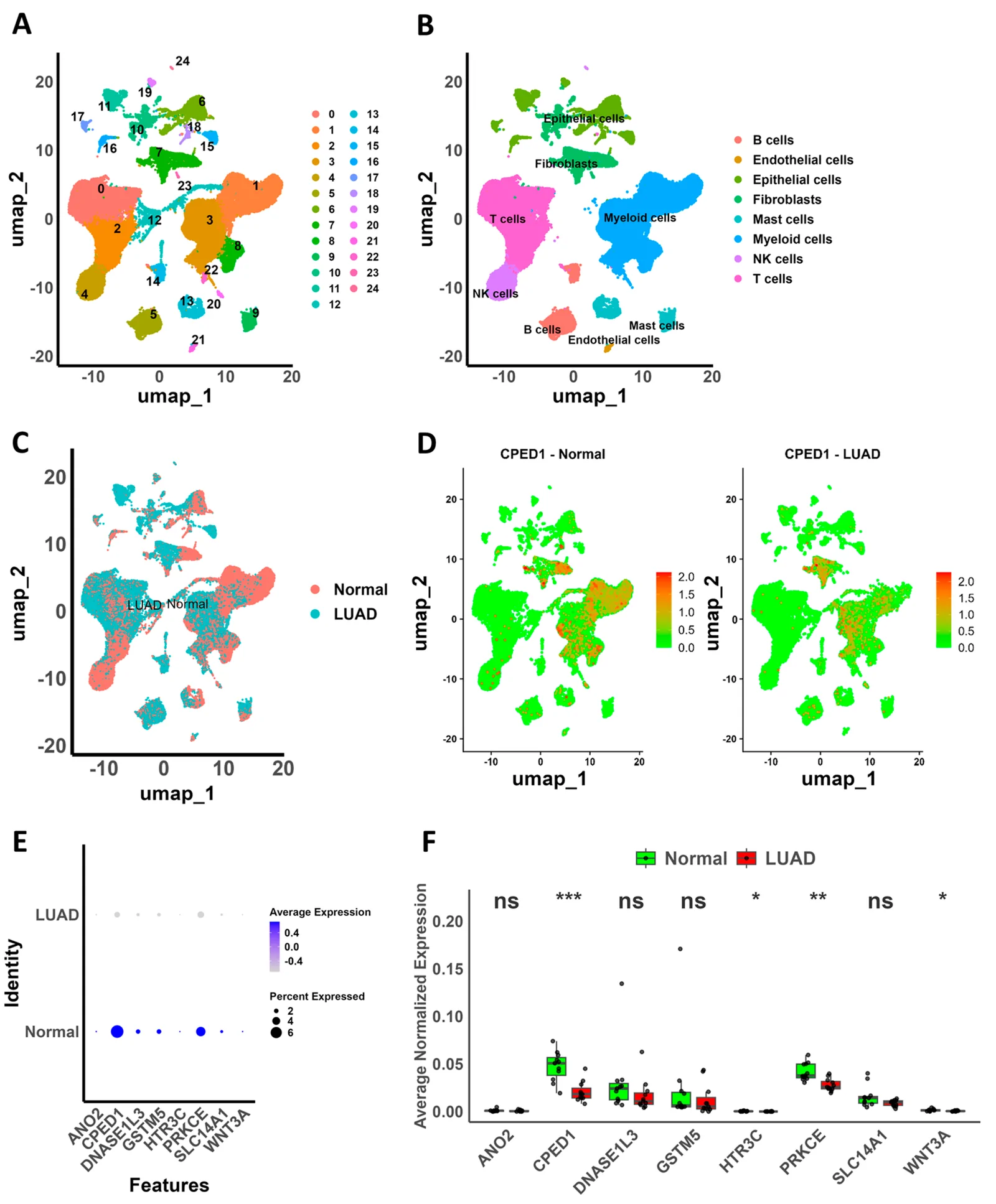
Single-cell transcriptomic landscape and gene expression in LUAD (*n* = 11) and normal samples (*n* = 11). (**A**) UMAP plots showing clustering of 25 transcriptional clusters. (**B**) Annotation of clusters into eight major cell types (B cells, endothelial cells, epithelial cells, fibroblasts, mast cells, myeloid cells, NK cells, and T cells). (**C**) UMAP plot comparing normal (red) and LUAD (blue) samples. (**D**) UMAP plots showing *CPED1* expression in normal and LUAD samples. (**E**) Dot plot showing average and percentage expression of eight candidate genes across cell populations. (**F**) Boxplots comparing average normalized expression in normal (green) and LUAD (red) samples. Significance was determined by Wilcoxon rank-sum test on sample-level averages with Benjamini–Hochberg FDR correction (ns = non-significant, * FDR < 0.05, ** FDR < 0.01, *** FDR < 0.001).

The DimHeatmap (Figure S3A) highlighted distinct gene loading patterns across PCs. The JackStraw plot (Figure S2H) confirmed statistical significance for the first 20 PCs, with *p*-values ranging from 8.02 × 10^−320^ (PC1) to 5.5 × 10^−29^ (PC20). The ElbowPlot (Figure S2I) showed a clear inflection at PC = 20, consistent with the cumulative variance plot (Figure S2J). Together, these approaches established 20 principal components as optimal for downstream clustering and visualization. UMAP clustering successfully grouped the single-cell samples into 25 clusters (Figure 10A). A total of 25,435 genes were used as marker genes for 25 cell clusters, and the top 20 significantly differentially expressed marker genes of each cluster are shown in a heatmap (Figure S3B). These 25 clusters were annotated into eight cell types: B cells, endothelial cells, epithelial cells, fibroblasts, mast cells, myeloid cells, NK cells, and T cells (Figure 10B). Dot plot analysis discovered distinct expression patterns: *CPED1*, *DNASE1L3*, *GSTM5*, and *PRKCE* showed broad expression, while *ANO2*, *HTR3C*, *SLC14A1*, and *WNT3A* were more restricted (Figure S2K). *CPED1* was expressed mostly in fibroblasts and myeloid cells, *DNASE1L3* in endothelial and mast cells, *GSTM5* in fibroblasts, and *SLC14A1* in mast cells, T cells, and NK cells (Figures S2L and S3C). Comparison of normal and LUAD samples highlighted clear separation of cell populations, highlighting LUAD-specific transcriptional states (Figure 10C). Pseudobulk analysis showed that *CPED1* expression was distinctly elevated in normal compared with LUAD samples (11 LUAD vs. 11 normal biological replicates), with enrichment in myeloid cells and fibroblasts (Figure 10D), and for the rest of the genes as shown in Figure S4. Dot plot analysis established higher average and percent expression of *CPED1*, *DNASE1L3*, *GSTM5*, and *PRKCE* in normal samples (Figure 10E). Boxplot analysis of sample-level averages showed that candidate genes (*CPED1* and PRKCE) were significantly downregulated in LUAD compared with normal samples (Wilcoxon rank-sum test, FDR < 0.05; Figure 10F).

### 3.10. Prognostic and Diagnostic Significance of CPED1 in LUAD

The cadherin-like and PC-esterase domain-containing protein 1 (*CPED1*) gene was strongly associated with survival risk in the prognostic nomogram. Also, patients with higher *CPED1* expression exhibited significantly better survival outcomes in LUAD samples (HR = 0.58, *p* = 2.3 × 10^−13^). It was observed that *CPED1* was the most frequently mutated gene in LUAD, with a mutation frequency of 41% and missense mutations detected in 47% of samples, followed by recurrent amplifications and deletions. In combined Enet (α = 0.1) and PLS diagnostic models, *CPED1* was found to be the strongest predictor. The validation of *CPED1* expression levels in independent bulk-RNA datasets confirmed significant downregulation in LUAD samples, with excellent diagnostic performance (AUC = 0.980 in GSE43458 and AUC = 0.885 in GSE31210). Finally, single-cell transcriptomic analysis demonstrated that *CPED1* expression was significantly lower in LUAD compared to normal samples, with enrichment predominantly in myeloid cells and fibroblasts. Collectively, these findings establish *CPED1* as a promising candidate biomarker, with both diagnostic and prognostic relevance in LUAD.

## 4. Discussion

Lung adenocarcinoma is characterized by its high aggressiveness, heterogeneity, and poor prognosis. Regardless of progress in chemotherapy, immunotherapy, and targeted therapies, the treatment of LUAD still faces challenges regarding treatment efficiency and prognosis [20]. Therefore, the identification of clinically appropriate prognostic biomarkers is necessary to advance personalized diagnosis and treatment.

This study combines multi-omics analyses, survival modeling, immune profiling, and machine learning to identify prognostic biomarkers in lung adenocarcinoma (LUAD). The findings highlight *CPED1* as a particularly promising candidate biomarker, consistently emerging across differential expression, mutation profiling, machine learning, and single-cell RNA sequencing analyses. Its downregulation in LUAD compared to normal tissues, combined with its high mutation frequency, underscores its potential role in tumorigenesis and disease progression.

To achieve this, we used TCGA-LUAD data consisting of 539 LUAD and 59 normal samples. A total of 5581 DEGs, including 2511 upregulated and 3070 downregulated genes, were identified using differential gene expression analysis. WGCNA identified the turquoise module (Corr = −0.79, *p* < 2.2 × 10^−308^) as strongly correlated with LUAD (*n* = 298). The intersection yielded 281 genes, which were then subjected to univariate Cox regression analysis, identifying 86 significant genes. Then, we identified a nine-gene signature (*ANO2*, *CHIAP2*, *CPED1*, *DNASE1L3*, *GSTM5*, *HTR3C*, *PRKCE*, *SLC14A1*, and *WNT3A*) via LASSO Cox regression analysis. Kaplan–Meier curves confirmed significantly worse survival in high-risk patients (log-rank *p* = 0.0027), and multivariate Cox regression established that risk score is an independent prognostic factor beyond stage and gender. ROC analyses showed moderate predictive accuracy, with AUCs of 0.672, 0.632, and 0.625 at 1, 3, and 5 years, respectively, consistent with prior LUAD prognostic models that achieved similar ranges [21]. Importantly, the nomogram provides a clinically interpretable tool for individualized survival prediction, echoing earlier reports where gene-based models improved patient classification. Notably, *CPED1*, *PRKCE*, and *SLC14A1* emerged as protective factors, while *HTR3C* was associated with poor prognosis. These findings are consistent with previous studies emphasizing the utility of multi-gene panels in classifying LUAD patients [22].

Our mutation analysis revealed that *CPED1* carries the highest mutation frequency among candidate genes in LUAD, with 41% of samples harboring mutations. Among all *CPED1* mutation events, missense mutations were the predominant type (47%). This aligns with reports that recurrent mutations in LUAD contribute to tumor heterogeneity and therapeutic resistance [3]. The amplification of *CPED1* and *WNT3A*, coupled with deletions in *DNASE1L3* and *SLC14A1*, highlights the diverse genomic mechanisms driving LUAD progression. Therefore, we can hypothesize that missense mutations in *CPED1* may be involved in causing mutations of target genes of anticancer drugs in LUAD, leading to drug resistance in cancer cells. However, the functional characteristics of missense mutations in *CPED1* have not been fully characterized in LUAD.

Pathway enrichment analysis revealed significant enrichment of nucleocytoplasmic transport, oxidative phosphorylation, protein processing in the endoplasmic reticulum, ribosome, and ribosome biogenesis in high-risk LUAD patients, based on FDR-adjusted values. Rather than implying direct pathway activation, these findings suggest that transcriptional dysregulation in these processes may contribute to poor prognosis, although functional validation is required to establish mechanistic links. Nucleocytoplasmic transport plays a critical role in lung cancer; dysregulated nuclear export, particularly through XPO1/RAN, promotes oncogenic signaling, metabolic reprogramming, and therapy resistance in LUAD [23]. Enhanced oxidative phosphorylation has been associated with aggressive LUAD, supporting tumor proliferation, metastasis, and poor survival, and has been incorporated into independent prognostic signatures [24]. Persistent stress and altered protein processing in the endoplasmic reticulum activate the unfolded protein response, which facilitates LUAD progression, immune evasion, and chemoresistance [25]. Elevated ribosome biogenesis and ribosomal activity are required to sustain the high translational demand of rapidly proliferating LUAD cells; multiple recent studies demonstrate that ribosome-biogenesis-related genes drive tumor growth, correlate with adverse prognosis, and represent promising therapeutic targets [26,27]. Collectively, the combined enrichment of these pathways in the high-risk group reflects a transcriptional program that favors uncontrolled growth, metabolic adaptation, and stress tolerance features that likely underlie the poor clinical outcomes observed in these patients.

In our present study, M1 macrophages were found to be significantly higher in the high-immune-score group. However, whether M1 macrophages were regulated by the identified pathways requires further investigation. Activated dendritic cells (aDCs), monocytes, and CD4^+^ naïve T cells showed statistical correlations with M1 macrophages in LUAD, underscoring macrophages as central hubs of immune regulation. CXCL9/10-DC expression has shown a positive correlation with infiltrating macrophages, specifically the M1 subtype [28], which is corroborated by our study. Monocytes, as precursors of macrophages, contribute directly to M1 polarization under inflammatory cues, reinforcing the antitumor phenotype [29]. In LUAD, the correlation between M1 macrophages and CD4^+^ naïve T cells is typically weak because M1 macrophages act on differentiated effector and memory T cells rather than maintaining a naïve state, and deconvolution algorithms often detect low baseline levels of truly naïve CD4^+^ cells in solid tumors [30]. Collectively, these interactions highlight a macrophage-centered immune network in LUAD, where M1 macrophages orchestrate cross-talk with dendritic cells, monocytes, and T cells to shape the tumor immune landscape.

Machine learning models, particularly combined Elastic Net (α = 0.1) and Partial Least Squares, confirmed *CPED1* as a key predictor. *CPED1* was also successfully validated in two GEO datasets and achieved high diagnostic accuracy across independent GEO datasets (AUCs up to 0.980). These results parallel other machine learning-based biomarker studies in LUAD, which have demonstrated the power of computational approaches in refining diagnostic tools [22]. Importantly, the reproducibility of *CPED1*’s predictive value across cohorts strengthens its candidacy as a promising biomarker, but it should be regarded as a hypothesis-generating finding that requires future functional and clinical validation.

Our single-cell RNA sequencing analysis revealed distinct cellular heterogeneity between LUAD and normal lung tissues, with *CPED1* markedly downregulated in tumor samples, particularly within fibroblasts and myeloid cells. This observation is consistent with prior single-cell studies that identified transcriptional reprogramming of fibroblasts and immune cells as key drivers of LUAD progression [16]. These findings align with prior single-cell studies showing that LUAD tissues exhibit altered fibroblast and myeloid cell transcriptional states, reshaping the tumor microenvironment [22]. Zhao et al. recently established a prognostic model using univariate regression and LASSO Cox regression with 16 genes, including *CPED1,* which showed significant downregulation in LUAD compared with normal samples and good predictive accuracy in LUAD prognosis [31], corroborating our findings.

Collectively, our findings highlight *CPED1* as a promising candidate biomarker for diagnosis and prognosis in LUAD. Its consistent performance across bulk and single-cell datasets, together with machine learning validation, suggests potential relevance and positions *CPED1* as a candidate for further functional and clinical investigation rather than as a definitive biomarker at this stage. Nonetheless, there are several limitations to this study. First, all analyses were computational and may not fully capture biological processes. Second, while machine learning and statistical analyses support *CPED1* as a diagnostic candidate, functional validation in vitro and in vivo is required. Third, the nine-gene prognostic model was developed and evaluated solely within the TCGA-LUAD dataset and currently lacks validation in independent external cohorts with long-term survival data; therefore, its generalizability and clinical utility remain to be established. Finally, although single-cell RNA sequencing provided valuable insights, the sample size and clinical diversity were limited, necessitating validation in larger and more diverse cohorts. Consequently, the prognostic signature and the role of *CPED1* should be regarded as preliminary and hypothesis-generating.

## 5. Conclusions

This comprehensive multi-omics and machine learning analysis highlights *CPED1* as a promising biomarker with potential diagnostic and prognostic relevance in LUAD. Mutation analysis revealed that *CPED1* carries the highest mutation frequency among candidate genes, with 41% of TCGA-LUAD samples harboring mutations. Among all *CPED1* mutation events, 47% were missense. CNV analysis further revealed frequent amplifications of *CPED1*. The nine-gene risk signature effectively stratified patients by survival outcomes within the TCGA cohort. Pathway enrichment analyses based on FDR-adjusted values revealed significant enrichment of nucleocytoplasmic transport, oxidative phosphorylation, protein processing in the endoplasmic reticulum, ribosome, and ribosome biogenesis in the high-risk group. Immune infiltration profiling highlighted macrophage-centered interactions, with M1 macrophages showing strong correlations with activated dendritic cells, monocytes, and CD4^+^ naïve T cells. Together, these findings provide new insights into LUAD molecular pathology, metabolic adaptation, and immune dynamics. Importantly, as this study is entirely computational and the prognostic model has not been validated in independent external survival cohorts, the findings should be regarded as preliminary and hypothesis-generating. Future functional experiments and multi-center prospective validation are essential before any consideration of clinical application.

## Data Availability

The data presented in this study, using the TCGA-LUAD and GSE43458, GSE31210, and GSE131907 datasets, are publicly available in the Genomic Data Commons (GDC, https://portal.gdc.cancer.gov, accessed on 30 June 2025) data portal and the NCBI Gene Expression Omnibus (GEO, http://www.ncbi.nlm.nih.gov/geo, accessed on 18 August 2025), respectively.

## Supplementary Materials

The following supporting information can be downloaded at: https://www.mdpi.com/article/10.3390/genes17091096/s1, Figure S1: Immune cell correlation heatmap (FDR-adjusted); Figure S2: Quality control and dimensionality reduction in single-cell RNA-seq data; Figure S3: Extended gene expression and dimensionality reduction analyses; Figure S4: UMAP plots showing candidate gene expression in normal and LUAD samples; Table S1: List of DEGs from TCGA-LUAD dataset (*n* = 5581); Table S2: List of identified genes in the turquoise module (*n* = 298; MM > 0.7, GS > 0.5); Table S3: The results of LASSO Cox regression analysis for nine genes; Table S4: Multivariate Cox regression analysis results.
