# Supplementary material for: Multi-Omics and Machine Learning Identify Immune-Linked Gene Signatures for LUAD Stratification
Source: Genes (Basel). 2026 Sep 11;17(9):1096. doi: 10.3390/genes17091096 (PMC13606826; doi:10.3390/genes17091096)
Supplement: Supplementary file 1 [file genes-17-01096-s001.zip › Supplementary Figures.pdf]

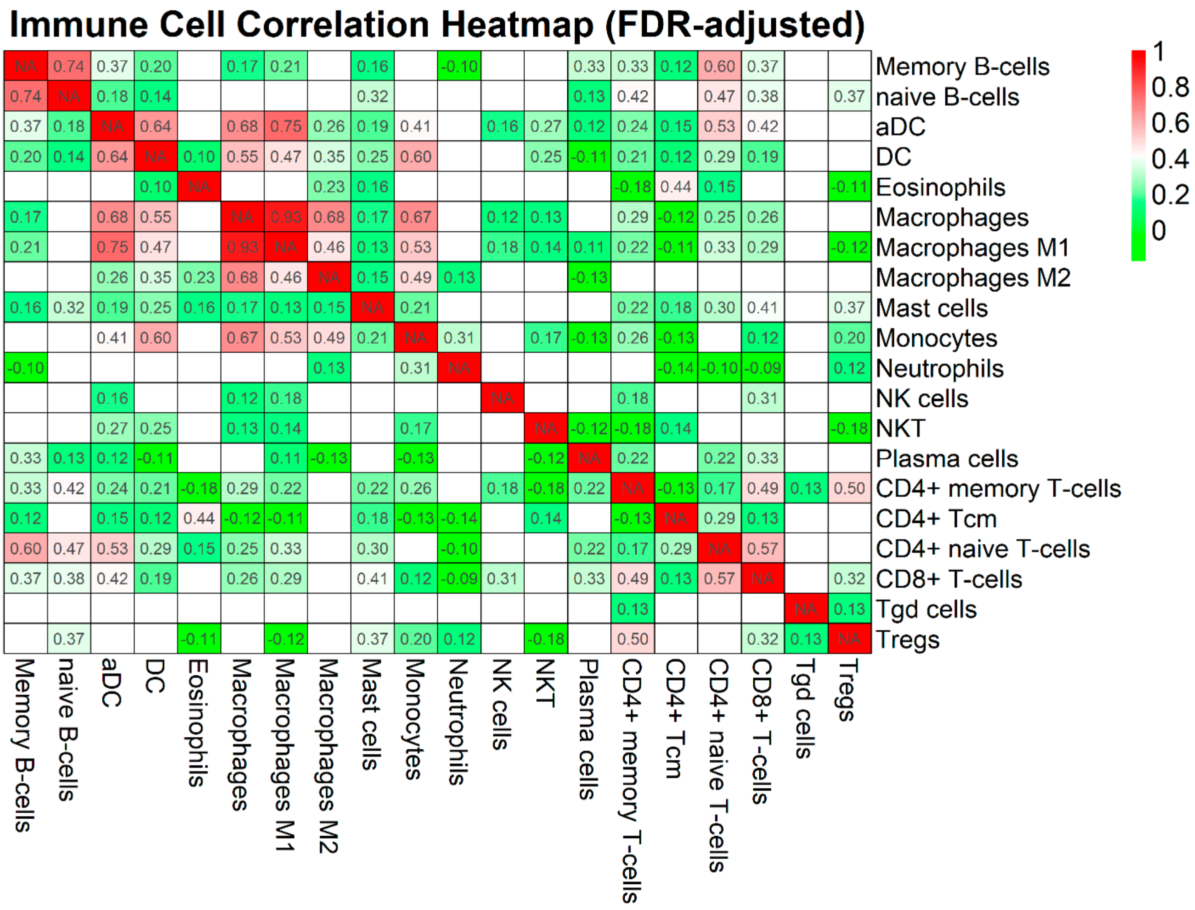

Figure S1. Immune cell correlation heatmap (FDR-adjusted).

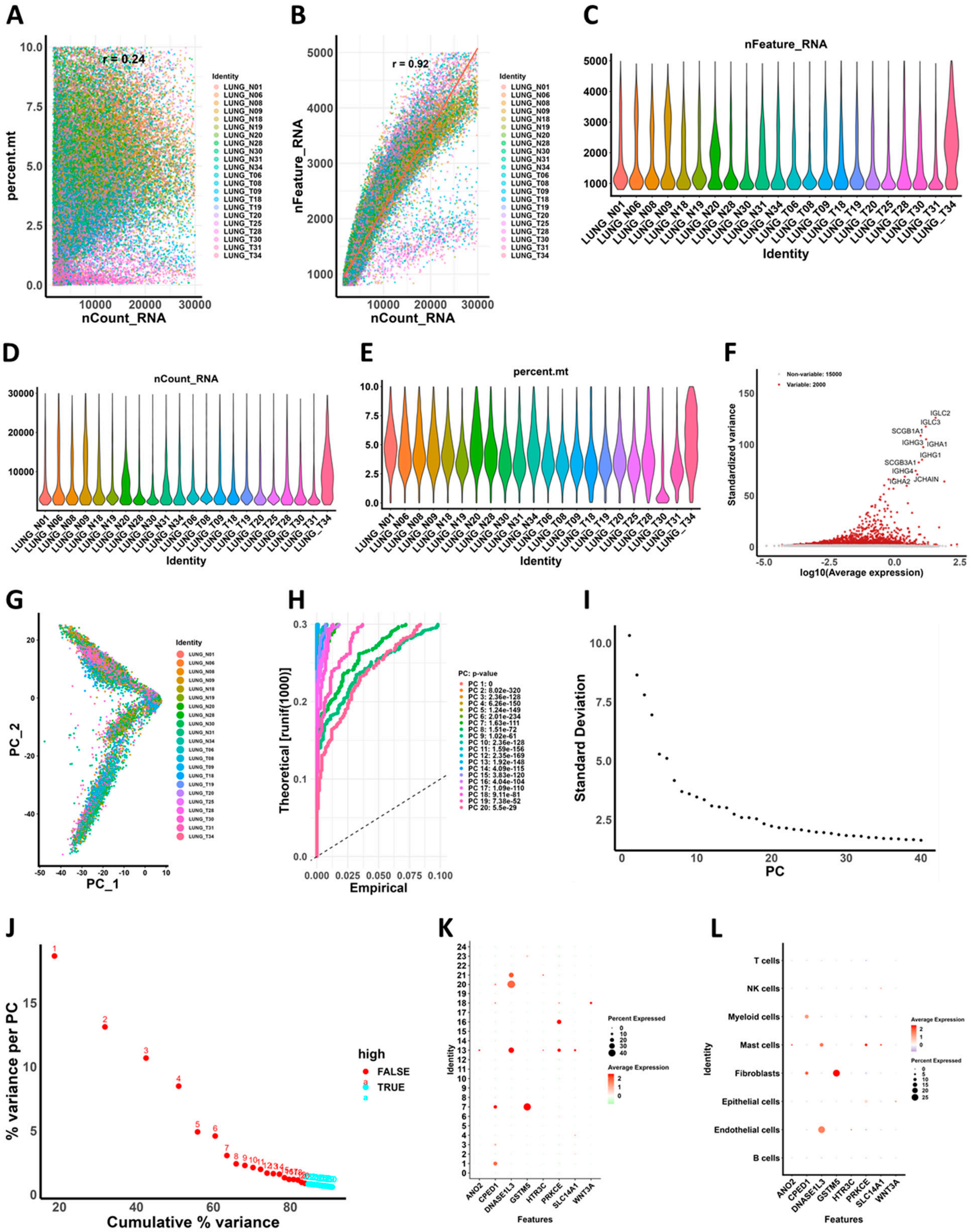
A
B
C
D
E
F
G
H
I
J
K
L
r = 0.24
percent.mt
nCount_RNA
Identity
r = 0.92
nFeature_RNA
nFeature_RNA
nCount_RNA
percent.mt
Non-variable: 15000
Variable: 2000
Standardized variance
log10(Average expression)
IGLC2
IGLC3
SCGB1A1
IGHG3
IGHA1
IGHG1
SCGB3A1
IGHG4
IGHA2
JCHAIN
PC_1
PC_2
Theoretical [runif(1000)]
Empirical
PC: p-value
PC 1: 0
PC 2: 8.02e-320
PC 3: 2.36e-128
PC 4: 6.26e-150
PC 5: 1.24e-149
PC 6: 2.01e-234
PC 7: 1.63e-111
PC 8: 1.51e-72
PC 9: 1.02e-61
PC 10: 2.36e-128
PC 11: 1.59e-156
PC 12: 2.35e-169
PC 13: 1.92e-148
PC 14: 4.09e-115
PC 15: 3.83e-120
PC 16: 4.04e-104
PC 17: 1.09e-110
PC 18: 9.11e-81
PC 19: 7.38e-52
PC 20: 5.5e-29
Standard Deviation
PC
% variance per PC
Cumulative % variance
high
FALSE
TRUE
Percent Expressed
Average Expression
Features
T cells
NK cells
Myeloid cells
Mast cells
Fibroblasts
Epithelial cells
Endothelial cells
B cells
ANO2
CPED1
DNASE1L3
GSTM5
HTR3C
PRKCE
SLC14A1
WNT3A

Figure S2. Quality control and dimensionality reduction in single-cell RNA-seq data. (A) Scatter plot of mitochondrial gene percentage (percent.mt) versus RNA counts (nCount_RNA), showing correlation across samples (r = 0.24). (B) Scatter plot of RNA features (nFeature_RNA) versus RNA counts (nCount_RNA), demonstrating strong correlation (r = 0.92). (C) Violin plots of nFeature_RNA across sample identities. (D) Violin plots of nCount_RNA across sample identities. (E) Violin plots of percent.mt across sample identities. (F) Scatter plot of standardized variance versus log10 average expression, highlighting highly variable genes. (G) PCA scatter plot (PC1 vs. PC2) showing sample clustering. (H) JackStraw plot comparing empirical versus theoretical p-values for principal components. (I) ElbowPlot showing standard deviation across principal components, used to determine optimal PC selection. (J) Cumulative variance explained by principal components, indicating proportion of variance captured (n = 20). (K) Dot plot of 8 candidate genes expression level across 25 transcriptional clusters. (L) Dot plot of 8 candidate genes across major cell types (T cells, NK cells, myeloid cells, mast cells, fibroblasts, epithelial cells, endothelial cells, B cells).

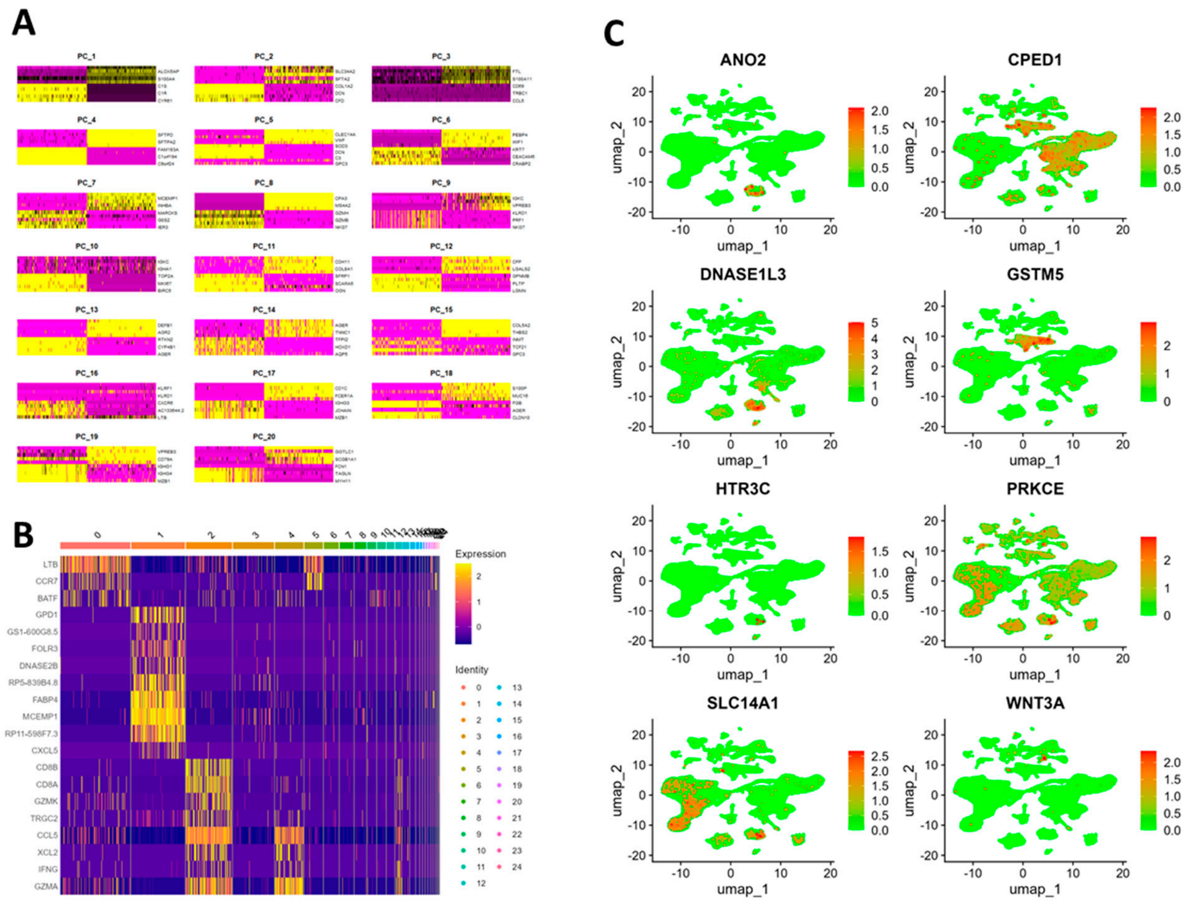

Figure S3. Extended gene expression and dimensionality reduction analyses. (A) Principal component loadings (PC1-PC20), illustrating gene contributions to variance in dimensionality reduction. (B) Clustered heatmap of gene expression across transcriptional clusters. (C) UMAP feature plots showing spatial expression of 8 candidate genes. Data points are colored by expression intensity (green = low, red = high).

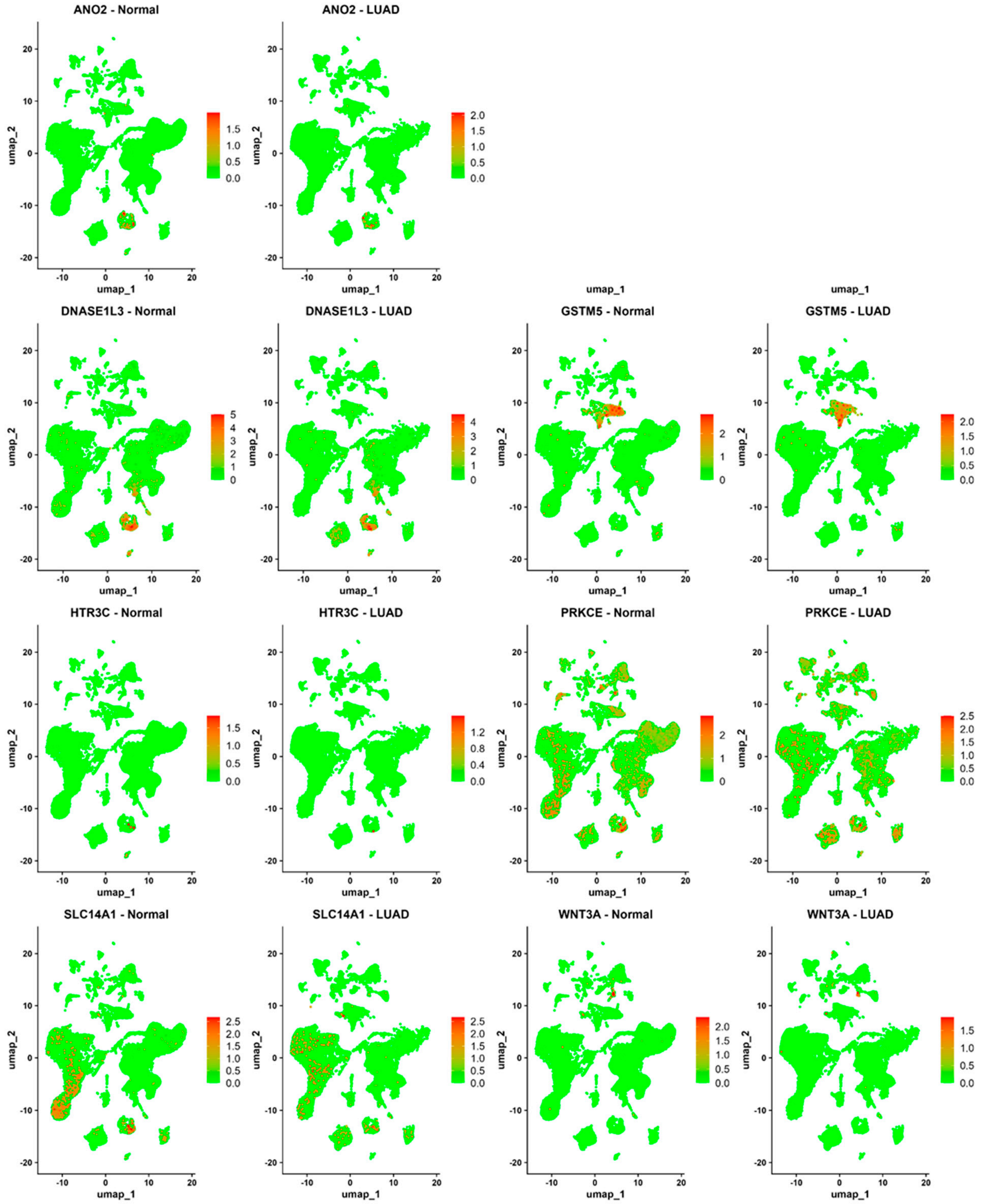

Figure S4. UMAP plots showing candidate gene expression in normal and LUAD samples
